# Impact of Sodium Alginate-Encapsulated Iron Nanoparticles and Soil Yeasts on the Photosynthesis Performance of *Lactuca sativa* L. Plants

**DOI:** 10.3390/plants13152042

**Published:** 2024-07-25

**Authors:** Daniela Berríos, Paola Fincheira, Felipe González, Christian Santander, Pablo Cornejo, Antonieta Ruiz

**Affiliations:** 1 Departamento de Ciencias Químicas y Recursos Naturales, Scientific and Technological Bioresource Nucleus BIOREN-UFRO, Universidad de La Frontera, Temuco 4811230, Chile; 2 Programa de Doctorado en Ciencias Agroalimentarias y Medioambiente, Facultad de Ciencias Agropecuarias y Medioambiente, Universidad de La Frontera, Temuco 4811230, Chile; 3 Laboratorio de Nanobiotecnología Ambiental, Centro de Excelencia en Investigación Biotecnológica Aplicada al Medio Ambiente (CIBAMA), Facultad de Ingeniería y Ciencias, Universidad de La Frontera, Av. Francisco Salazar 01145, Temuco 4811230, Chile; 4 Programa de Doctorado en Ciencias Mención Biología Celular y Molecular Aplicada, Facultad de Ciencias Agropecuarias y Medioambiente, Universidad de La Frontera, Temuco 4811230, Chile; 5 Escuela de Agronomía, Facultad de Ciencias Agronómica y de los Alimentos, Pontificia Universidad Católica de Valparaíso, Quillota 2260000, Chile; 6 Centro Regional de Investigación e Innovación para la Sostenibilidad de la Agricultura y los Territorios Rurales, CERES, La Palma, Quillota 2260000, Chile

**Keywords:** yeast, soil, nanoparticles, photosynthesis

## Abstract

In a scenario of accelerated global climate change, the continuous growth of the world population, and the excessive use of chemical fertiliser, the search for sustainable alternatives for agricultural production is crucial. The present study was conducted to evaluate the plant growth-promoting (PGP) characteristics of two yeast strains, *Candida guilliermondii* and *Rhodotorula mucilaginosa*, and the physicochemical characteristics of nanometric capsules and iron oxide nanoparticles (Fe_2_O_3_-NPs) for the formulation of nanobiofertilisers. The physiological and productive effects were evaluated in a greenhouse assay using lettuce plants. The results showed that *C. guilliermondii* exhibited higher tricalcium phosphate solubilisation capacity, and *R. mucilaginosa* had a greater indole-3-acetic acid (IAA) content. The encapsulation of *C. guilliermondii* in sodium alginate capsules significantly improved the growth, stomatal conductance, and photosynthetic rate of the lettuce plants. Physicochemical characterisation of the Fe_2_O_3_-NPs revealed a particle size of 304.1 nm and a negative Z-potential, which indicated their stability and suitability for agricultural applications. The incorporation of Fe_2_O_3_-NPs into the capsules was confirmed by SEM-EDX analysis, which showed the presence of Fe as the main element. In summary, this study highlights the potential of nanobiofertilisers containing yeast strains encapsulated in sodium alginate with Fe_2_O_3_-NPs to improve plant growth and photosynthetic efficiency as a path toward more sustainable agriculture.

## 1. Introduction

The continuous growth of the population has resulted in a growing demand for food, which constitutes one of the main challenges in agriculture [1]. Diverse studies have been performed to mitigate problems associated with climate change and increase crop production. Unfortunately, the application of conventional agrochemicals, such as chemical fertilisers and pesticides, have negatively affected the environment and the quality of food [2,3,4]. The low nutrient efficiency of fertilisers and the scarcity of water and available land accentuate problems in agriculture [5]. Therefore, various alternatives have been proposed to increase sustainability in agriculture and the production of chemical-free foods [6]. For example, the development of nanotechnology in agriculture has played an important role in recent years to improve the quality of food productivity [7].

Nanoparticles (NPs) are characterised by their small size, high surface-to-volume ratio, optical properties, controlled release kinetics at target sites, low-cost formulation, and greater biological activity [8,9,10]. The application of NPs as nanofertilisers has shown great effectiveness in increasing photosynthesis, nutrient and water uptake, secondary metabolism, antioxidant enzymatic activity, cell wall formation, and stress tolerance in some crops, such as common bean (*Phaseolus vulgaris*), maize (*Zea mays*), peanut (*Arachis hypogaea*), wheat (*Triticum aestivum*), coffee arabica (*Coffea arabica*), and spinach (*Spinacia oleracea*) [11,12,13,14,15,16]. Iron nanoparticles (Fe-NPs) have been synthesised for application in plants due to their relevant role in biochemical and physiological processes [2]. Many factors influence photosynthesis, including genetic factors and external environmental factors [17,18], such as light, temperature, CO_2_ concentration [19], hormones [20], and mineral elements [21]. All of these factors influence photosynthesis and increase the amount of CO_2_, which is crucial for photosynthesis and is the main source of carbon for the production of plant biomass and crop yield. Improvements in photosynthesis have a direct beneficial impact on crop yield [22,23].

Iron is a central co-factor of vital enzymes and participates in electron chains in plants. It is essential for photosynthesis and chlorophyll biosynthesis [24]. Fe-NPs have beneficial effects in plants depending on their concentration, crop variety, and plant contact time. Fe-NPs increased root length, leaf number, leaf area, and chlorophyll content in corn seedlings [13]. These same NPs improved the percentage of seed germination, seedling growth, and the physiological aspects of watermelon plants exposed to environmental stress [25,26]. α-Fe_2_O_3_-NPs increased the seed vigour index, shoot length, and weight of *Lycopersicum esculentum* plants [27]. Fe-NPs had beneficial effects on *Medicago falcata*, *Eruca sativa*, *Nicotiana tabacum*, and *Phaseolus vulgaris* via increases in soil nutrients, chlorophyll content, weight, and root length [28,29,30].

In addition to nanotechnology, soil microorganisms have been extensively investigated for their ability to promote plant growth, which makes them an important biotechnological tool [31]. Bacteria, mycorrhiza-forming fungi, actinomycetes, and yeasts effectively improved nutrient availability, modulated phytohormones pathways, and protected against pathogens [32]. Yeasts are one of the most abundant microorganisms in soil, but they have been poorly studied [33,34,35]. Yeasts are unicellular and polyphyletic fungi that can form meiospores on a basidium or within an ascus [36]. Yeast has potential as a plant growth-promoting (PGP) microorganism via different mechanisms, such as the production of organic acids, siderophores, phytohormones, and enzymes such as chitinase or 1-aminocyclopropane-1-carboxylate (ACC) deaminase; the enhancement of phosphate solubilisation; increasing the translocation of macro- and micronutrients; and improving photosynthetic activity and resistance to abiotic stress [37,38,39].

Sodium alginate is a polysaccharide with agricultural, food, and pharmaceutical applications. This polymer has been used to encapsulate microorganisms due to its biodegradability, biocompatibility, non-toxic nature, low cost, and stability in soils, which allows a slow and constant release of microorganisms [40]. Based on this background, in the present study we propose the encapsulation of Fe-NPs and PGP yeast in a sodium alginate-based matrix. We used lettuce (*Lactuca sativa*) as a model plant to determine the impact of Fe-NPs and yeast soil encapsulated in alginate-based capsules. Lettuce is a widely grown and consumed crop worldwide, and it is considered a healthy vegetable due to its content of phenolic compounds, vitamin C, folates, carotenoids, and other antioxidant compounds [41,42]. The yeasts used here were previously collected and were first studied by our group. Therefore, the present study hypothesised that the encapsulation of Fe-NPs with yeasts would have a positive effect on the photosynthesis of lettuce plants. The effect of encapsulation on the photosynthetic behaviour of lettuce plants was evaluated. This study was conducted to (1) determine the plant growth-promoting capacity of *Candida guilliermondii* and *Rhodotorula mucilaginosa*, (2) formulate beads based on sodium alginate containing commercial Fe_2_O_3_-NPs and yeasts, and (3) determine the effects on productive and photosynthetic traits by incorporating the formulated encapsulation in lettuce plants under greenhouse conditions.

## 2. Results

### 2.1. Determination of Plant Growth-Promotion (PGP) Traits of Yeast Strains

The PGP characteristics of two yeasts, *Candida guilliermondii* and *Rhodotorula mucilaginosa*, were evaluated (Table 1). Phosphate solubilisation was evaluated at 24 h and 14 days. Both strains reached the same phosphate solubilisation values at 24 h, but *Candida guilliermondii* solubilised 122% more phosphate than *Rhodotorula mucilaginosa* on day 14. *R. mucilaginosa* produced 28% more indole acetic acid (IAA) than *C. guilliermondii* at 7 days. The presence of siderophores and 1-aminocyclopropane-1-carboxylate (ACC) deaminase was positive for both strains, which indicated their production capacity.

### 2.2. Physicochemical Characterisation of Nanocapsules and Iron Oxide Nanoparticles (α-Fe_2_O_3_-NPs)

**Figure 1:**
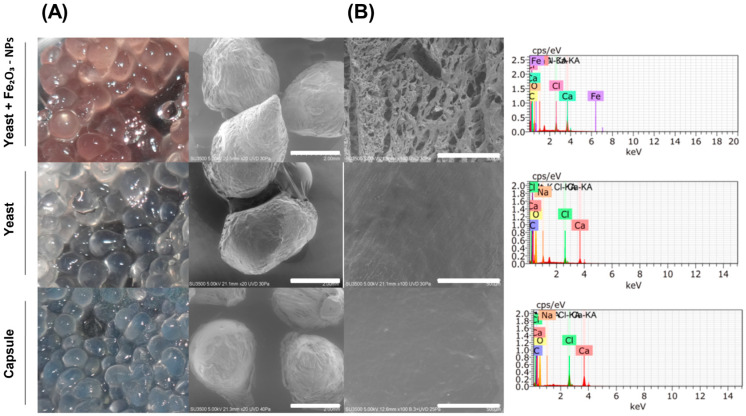
Morphological characterisation of sodium alginate encapsulates. From top to bottom: (**A**) Beads containing *Candida guilliermondii* yeast and α-Fe_2_O_3_ nanoparticles; capsule with yeast and capsule as carrier, each accompanied by representative photographs of the surface of the capsules. (**B**) Scanning electron microscopy coupled with X-ray elemental microanalysis (SEM/EDX). The white bars indicate the reference lengths of 2 mm and 500 µm, respectively.

The formulated capsules containing α-Fe_2_O_3_-NPs and *C. guilliermondii*, capsules containing only yeast, and capsules composed only of sodium alginate were also analysed (Figure 1A). All of these capsules showed an oval shape and homogeneous surface, but a change in tonality was observed when nanoparticles were added and resulted in a light brown colour.

SEM-EDX analysis (Figure 1B) confirmed the incorporation of Fe_2_O_3_-NPs into the capsules with Fe as the main element, and other elements, such as Ca, Cl, O, C, and Na, were present due to the sodium alginate and calcium chloride used for the formulation of the capsules. Fe was not detected in the other two capsules.

**Figure 2:**
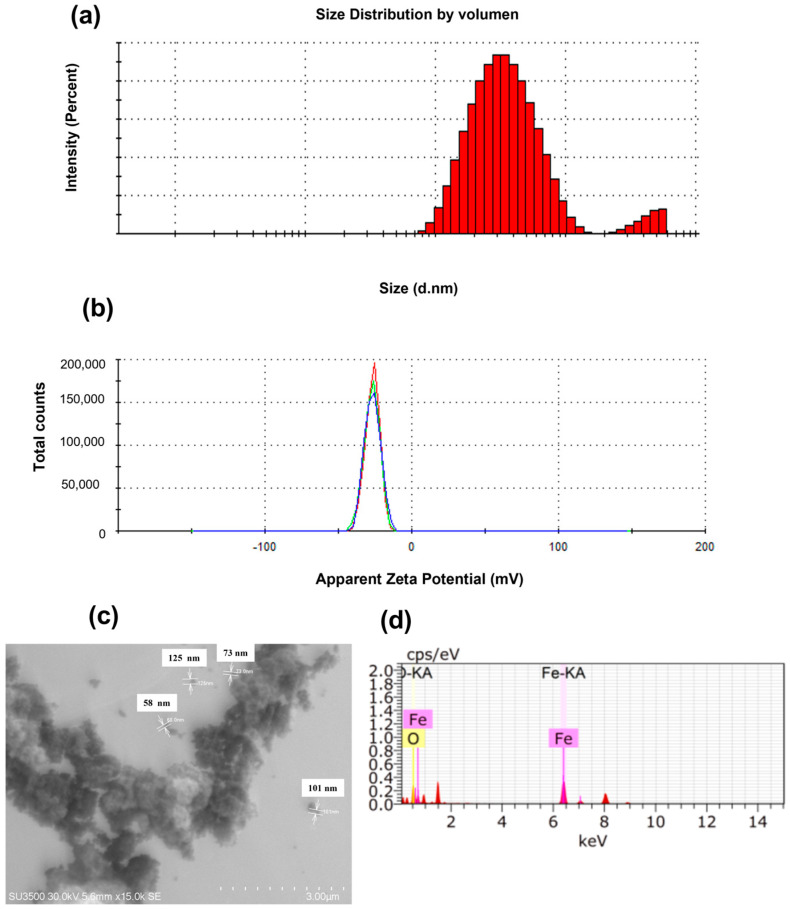
Physicochemical characterisation of α-Fe_2_O_3_ nanoparticles. The determinations were as follows: (**a**) nanoparticle size distribution, (**b**) zeta potential measured by dynamic light scattering, (**c**) representative photograph captured by scanning transmission electron microscopy (STEM), and (**d**) elemental composition of the surface.

The mean hydrodynamic particle size of the Fe_2_O_3_-NPs was 304.1 nm, with a polydispersity index of 0.27, which indicates the high homogeneity of these particles (Figure 2a). The Z-potential of −26.7 mV indicated the stability of the Fe_2_O_3_-NPs, which is an important characteristic for its agricultural application (Figure 2b). SEM examination of the Fe_2_O_3_-NPs revealed a spherical shape in the solid state and a mean size of 89.25 nm (Figure 2c). The elemental composition measured by SEM/EDX confirmed the presence of Fe and O (Figure 2d).

### 2.3. Biomass Production of Lettuce Plants

**Figure 3:**
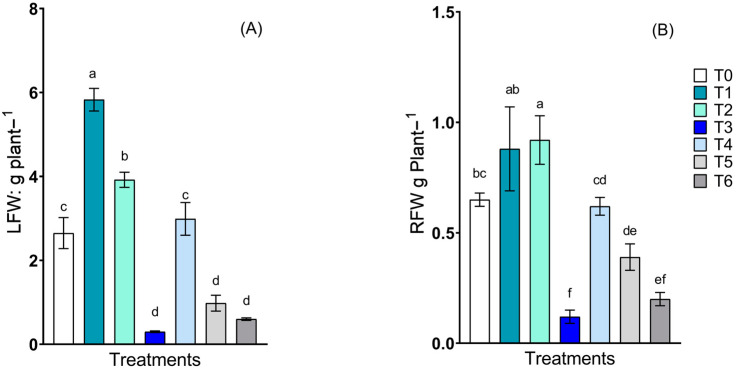
Biomass production in lettuce plants inoculated with *Candida guilliermondii*, *Rhodotorula mucilaginosa* and a consortium, in the presence and absence of nanoparticles by means of alginate nano-encapsulates. Fresh leaf weights (LFW) and fresh root weights (RFW). The treatments described were as follows: control non-inoculated (T0); *C. guilliermondii* with NPs (T1); *C. guilliermondii* without NPs (T2); *R. mucilaginosa* with NPs (T3); *R. mucilaginosa* without NPs (T4); consortium with NPs (T5); and consortium without NPs (T6). Values are means of four replicates ± S.E. Bars sharing the same lowercase letters between treatments are not significantly different according to Tukey multiple range test (*p* < 0.05).

Significant differences in leaf fresh weight were detected between treatments (Figure 3A), which was primarily observed for *C. guilliermondii* treated with NPs. This group exhibited a 120% increase in growth compared to the non-inoculated control and a 32.7% increase compared to the same yeast strain but without NPs. The *R. mucilaginosa* with NPs and both consortia with and without NPs treatments (T3, T5, and T6, respectively) exhibited the lowest growth compared to the control treatment. For root growth (Figure 3B), significant differences were observed between the treatments. T2 exhibited greater growth, which was 41.5% greater than the control treatment, but there were no significant differences from T1, which corresponded to the same yeast strain. Treatments T3, T5, and T6 followed the same trend observed for leaf growth.

### 2.4. Photosynthesis and Water Status

**Figure 4:**
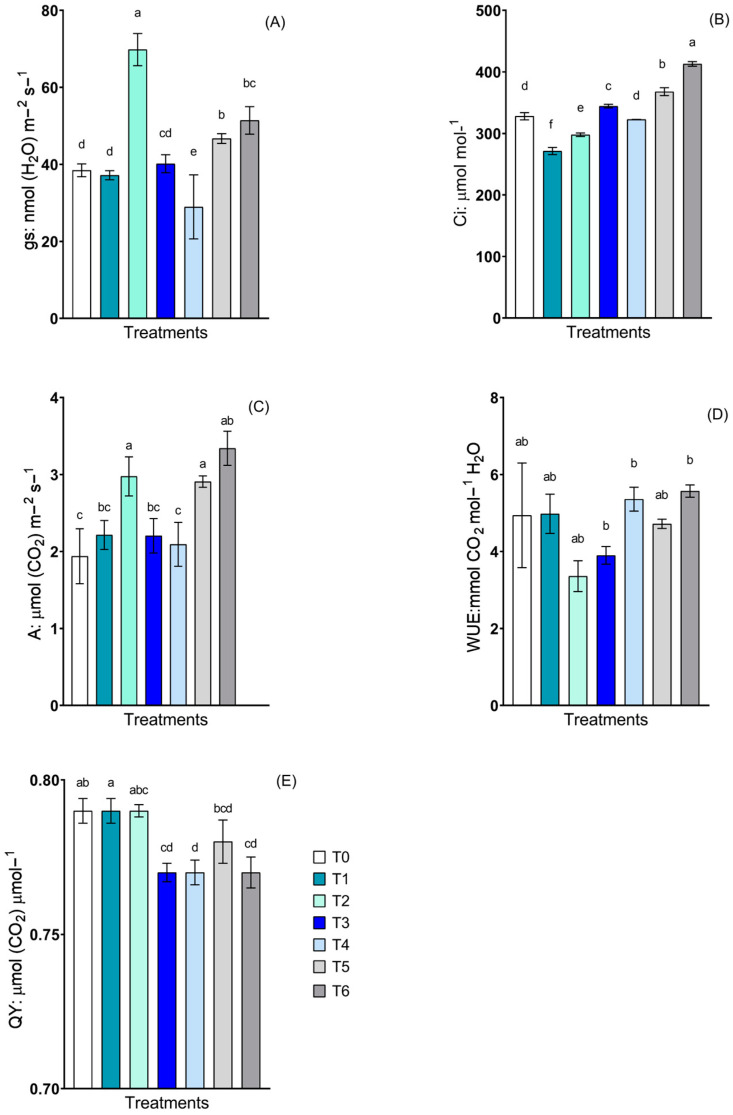
Determinations of photosynthetic characteristics and water use efficiency in lettuce plants inoculated with *Candida guilliermondii*, *Rhodotorula mucilaginosa* and a consortium, in the presence and absence of nanoparticles. (**A**) Stomatal conductance (gs); (**B**) internal CO_2_ concentration in leaves (Ci); (**C**) photosynthetic rate (A); (**D**) water use efficiency (WUE); (**E**) quantum yield of photosystem II (QY). The treatments described were as follows: control non-inoculated (T0); *C. guilliermondii* with NPs (T1); *C. guilliermondii* without NPs (T2); *R. mucilaginosa* with NPs (T3); *R. mucilaginosa* without NPs (T4); consortium with NPs (T5); and consortium without NPs (T6). Values are means of four replicates ± S.E. Bars sharing the same lowercase letters between treatments are not significantly different according to Tukey multiple range test (*p* < 0.05).

Photosynthetic characteristics, such as stomatal conductance (gs), internal CO_2_ concentration (Ci), photosynthetic rate (A), water use efficiency (WUE), and photosystem II efficiency (QY), were evaluated (Figure 4). All parameters showed noticeable differences between treatments, except WUE, where statistically significant differences were detected.

Treatment T2 had the greatest gs (Figure 4A) of 69.8 nmol (H_2_O) m^−2^ s^−1^, which was 82% greater than the control treatment. The Ci (Figure 4B) differed between all treatments and the control, except T4. Trends in decreasing Ci were observed for the treatments containing the *C. guilliermondii* strain (T1, T2), except the plants inoculated with the consortium (T6, T7), where increases in Ci concentrations were observed. T6 and T7 were 12.16% and 26% greater than the non-inoculated control treatment (T0), respectively.

*C. guilliermondii* without NPs and consortium with NPs showed the highest A (Figure 4C), compared to the control. Regarding QY (Figure 4E), *R. mucilaginosa* with and without NPs and the consortium without NPs exhibited statistically significant differences compared to the non-inoculated control, with an efficiency less than 2.53% in all treatments compared to T0.

### 2.5. Photosynthetic Pigments

**Figure 5:**
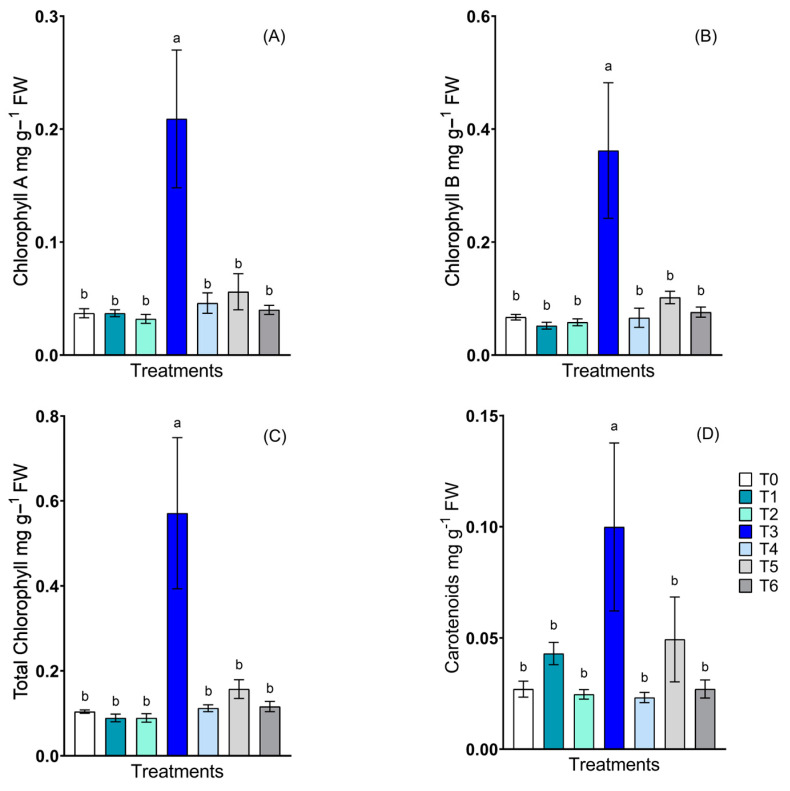
Determinations of photosynthetic pigments in lettuce plants inoculated with *Candida guilliermondii*, *Rhodotorula mucilaginosa* and a consortium, in the presence and absence of nanoparticles. (**A**) Chlorophyll A; (**B**) chlorophyll B; (**C**) total chlorophyll; and (**D**) carotenoids. The treatments described were as follows: control non-inoculate (T0); *C. guilliermondii* with NPs (T1); *C. guilliermondii* without NPs (T2); *R. mucilaginosa* with NPs (T3); *R. mucilaginosa* without NPs (T4); consortium with NPs (T5); and consortium without NPs (T6). Values are means of four replicates ± S.E. Bars sharing the same lowercase letters between treatments are not significantly different according to Tukey multiple range test *p* < 0.05.

The contents of chlorophyll and carotenoids revealed similar patterns (Figure 5). For the contents of chlorophyll A (Figure 5A), chlorophyll B (Figure 5B), total chlorophyll (Figure 5C), and total carotenoids (Figure 5D), *R. mucilaginosa* treatment without NPs was the only treatment that demonstrated statistically significant differences compared to the control treatment, with increases in activity of 465%, 440%, 449%, and 267%, respectively.

### 2.6. Multivariate Analysis 

**Figure 6:**
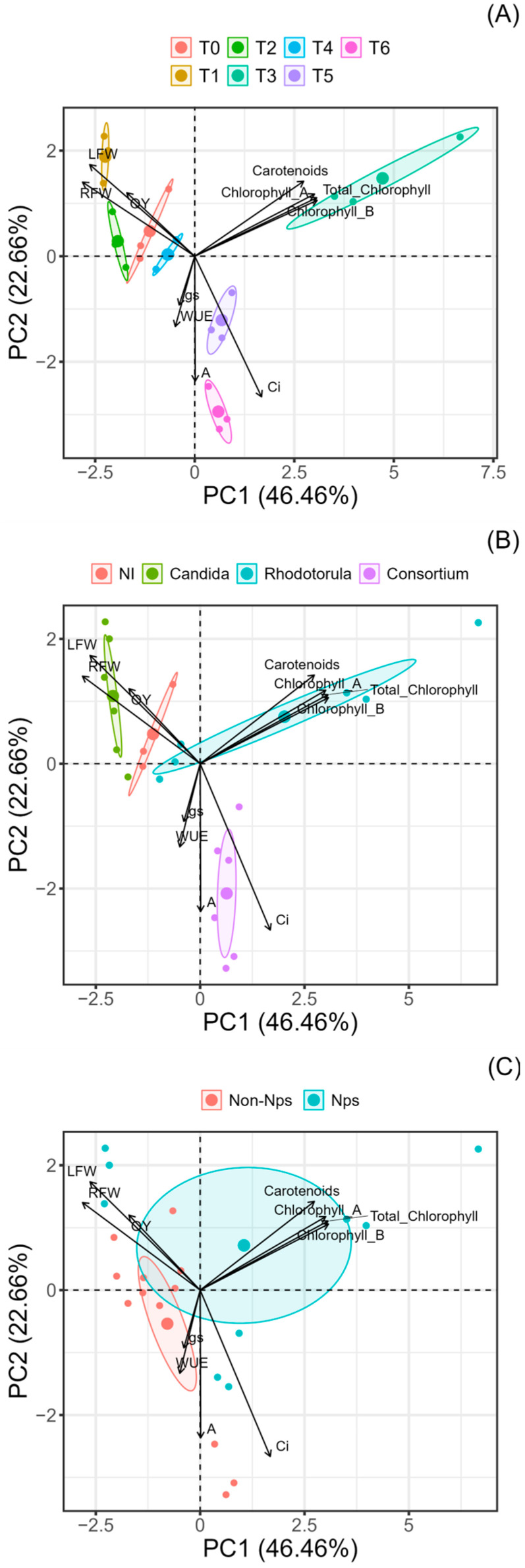
Principal component analysis (PCA) considering the overall behaviour of the different treatments (**A**); the evaluation of the different inoculant used (**B**); and the presence or absence of Fe_2_O_3_ nanoparticles (**C**). The treatments described were as follows: control non-inoculate (T0); *Candida guilliermondii* with NPs (T1); *Candida guilliermondii* without NPs (T2); *Rhodotorula mucilaginosa* with Nps (T3); *Rhodotorula mucilaginosa* without NPs (T4); consortium with NPs (T5); and consortium without NPs (T6). Fresh weight leaves (LFW); fresh weight roots (RFW); stomatal conductance (gs); internal CO_2_ concentration in leaves (Ci); photosynthetic rate (A); water efficiency (WUE); quantum yield of photosystem II (QY); chlorophyll A; chlorophyll B; total chlorophyll; carotenoids.

Factorial analysis using the principal components showed that 69.12% of the total variance was explained for principal component 1 (PC1) (44.46%) and principal component 2 (PC2) (22.66%). The behaviour of the treatments in relation to each of the experimental determinations was evaluated (Figure 6A). Associations of the *R. mucilaginosa* treatment with NPs were observed with the determinations of chlorophylls A and B, and total chlorophylls and carotenoids. In addition, *C. guilliermondii* with NPs treatment positively correlated with growth traits in leaves and roots. The associations of the different inoculums applied and the different experimental variables were also evaluated (Figure 6B). Treatment with the *C. guilliermondii* strain was positively associated with leaf and root fresh weight, and treatment with the *R. mucilaginosa* strain was associated with chlorophyll and carotenoid concentrations. The consortium treatments were associated with two photosynthetic treatments, A and Ci. For the NPs (Figure 6C), the presence of nanoparticles was associated with several parameters, such as chlorophyll, carotenoids, leaf weight root, and QY, and the absence of nanoparticles was associated with some parameters of photosynthesis, such as gs, WUE, and A.

For all the variables studied, a two-way analysis of variance (ANOVA) was performed, considering the yeast, the nanoparticle, and the interaction between both variables as the source of variation. The results showed significance in several of the interactions evaluated; the presence of yeast showed significant effects (*p* < 0.0001) in the variables chlorophyll A, chlorophyll B, Ci, and gs. The nanoparticles significantly influenced (*p* < 0.001) the variables of total chlorophyll and carotenoids. The interaction between yeast and nanoparticles was significant (*p* < 0.05) in the variables of carotenoid production, chlorophyll A, B, and WUE (Table S1).

## 3. Discussion

PGP demonstrated that the *C. guilliermondii* and *R. mucilaginosa* yeast strains exerted favourable growth-promoting effects on plants for all of the evaluated characteristics. Although both strains showed PGP capabilities, the *C. guilliermondii* strain exhibited a greater phosphate solubilisation capacity, which is consistent with Silambarasan et al. [43] who demonstrated that the *Candida* sp. strain solubilised 1.17 mg mL^−1^ tricalcium phosphate. These findings suggest that yeasts play an active role as facilitators of the supply of phosphate to plants, which is directly related to greater plant growth, because siderophores bind to the unavailable form of Fe³⁺ to form stable complexes that make it available for uptake and assimilation by plants in different processes, which results in greater growth [37]. Another important characteristic is the production of IAA. Although both yeast strains produce IAA, *R. mucilaginosa* exhibited greater production. Silambarasan et al. and Duca and Glick [43,44] demonstrated that different soil microorganisms, yeasts, bacteria, or actinobacteria produced IAA from an L-thiophane precursor. This phytohormone is important because it participates in the regulation of plant development, and it is directly related to growth [45]. Although the production of siderophores and ACC deaminase was positive in both strains, these parameters depend on other factors, such as the type of crop, the type of microorganisms, and their interaction with the soil–plant system [46].

The use of sodium alginate beads as carriers for yeasts offers several advantages, including protection during storage and post-application [47,48] and improvement in the shelf life of formulations [49,50]. Therefore, these beads represent a viable alternative for the encapsulation of PGP microorganisms and maintain biological effectiveness under field conditions [51]. The procedure proposed in the present study allows the encapsulation of more than one system, in our case a yeast and a nanoparticle, to offer a controlled release of nutrients (iron), which facilitates direct application to improve certain plant characteristics, such as photosynthesis, antioxidant response, and productive traits, included as the traits reflected in the present study [26,52].

The NPs used were characterised, and the results showed a particle size of 304.1 nm, which is different from Lu et al. [53], who reported a particle size of 125 nm. This difference may be due to the synthesis procedure of the NPs. However, the Z potential was negative in both studies, which indicated a degree of stability that supports its potential use in agricultural applications, as demonstrated in the present study. According to the typical definition of nanoscale, nanomaterials (NMs) are materials with building units between 1 and 1000 nm in at least one dimension [54]. Although the size of the NPs is an important factor when applying a nanobiofertiliser, there are other factors that influence the applicability of this type of nanomaterials such as the shape of the NPs, the surface, the area, and the morphology, which are parameters that will allow evaluating the specific zone of interaction between the plant structures and the NPs. It is also important to mention that the Z-potential is another important parameter, since the attachment of the NPs to the plant cell surface depends directly on the charges of the NPs, and the state of dispersion of the NPs in aqueous solution influences the application routes [55,56].

The biomass of the lettuce plants showed that the PGP traits of *C. guilliermondii*-treated plants exhibited improved growth, which may be due to its ability to produce different types of siderophores, IAA, SA (salicylic acid), and DHBA (2,3—dihydroxybenzoic acid), which are different from *R. mucilaginosa*, as reported by Silambarasan et al. [37,43]. Notably, these characteristics play an essential role in the micronutrient nutrition of yeasts and growth [57,58,59]. Previous studies using *C. tropicalis* yeast as an inoculum on rice (*Oriza sativa*) seedlings revealed increases in dry weight of up to 35% compared to non-inoculated seedlings. Sarabia et al. [31] studied maize (*Zea mays*) plants inoculated with different yeast strains and showed significant increases in shoot and root weights compared to non-inoculated plants. Fernández San-Millán et al. [38] also reported increases in shoot and root biomass in *Nicotiana benthamiana* seedlings inoculated with different yeast strains. All of these reports are consistent with our results, primarily in terms of the ability of *C. guilliermondii* over *R. mucilaginosa* to favour the growth of lettuce plants. Although the role of these yeasts in plant growth is understood, the presence of nanoparticles was also a determining factor in this study because T1 contained Fe-NPs. This result may be explained by the ability of siderophores to improve iron uptake by plants [60].

Photosynthetic activity plays a pivotal role in agriculture, particularly in enhancing crop productivity and biomass [22]. Regarding the evaluated photosynthetic traits, gs and Ci were crucial because these characteristics exhibited high values in treatments inoculated with *C. guilliermondii* plus the Fe-NPs compared to the control treatment and the treatments containing the *R. mucilaginosa* yeast (T1 and T3). This difference may be directly related to an increase in the A due to a greater stomatal aperture [61]. Similar results were obtained by Shah et al. [62], who reported that the application of iron oxide NPs improved the photosynthetic rate of melon (*Cucumis melo*) plants subjected to abiotic stress due to the faster entry of CO_2_, which increased the efficiency of photosynthesis.

The present study revealed no variation in WUE; some studies suggest that this may be due to the increased carboxylase efficiency of RuBisCo compared to its oxygenase activity, a decrease in photorespiration and increase in carbon uptake, which are beneficial for plant growth [63,64]. However, higher assimilation rates tend to decrease stomatal conductance, primarily because the increased amount of CO_2_ entering the plant is fixed faster in sugars [47]. Another important aspect to consider in our results is the presence or absence of iron nanoparticles. Iron is essential for plant growth and photosynthetic processes, primarily photosynthetic efficiency, because it forms part of the structure of photosynthetic tissues as a constituent of complexes that involve electron transport [65]. This factor is important because it suggests that the treatments did not exhibit differences between themselves, and some plants even showed unsuccessful photosynthesis.

The photosynthetic pigments, chlorophylls A and B, and total chlorophyll and carotenoids did not directly influence photosynthesis and did not exhibit significant differences, except for the *R. mucilaginosa* treatment without NPs. Although there are no previous studies using yeast as an inoculant, there are studies of bacteria with PGP capacities in which the concentration of pigments was preferentially responsive to some specific type of microorganism. For example, Santander et al. [66] reported that an increase in chlorophyll concentration occurred with the use of two of the eight bacterial inoculants studied. Nevertheless, the carotenoid content did not differ between the eight different bacteria in the lettuce plants, which is similar to the results of the present study.

Multivariate PCA revealed that the *C. guilliermondii* strain was positively linked to increased leaf and root growth, while the *R. mucilaginosa* strain responded to increasing photosynthetic pigments, which suggests that the responses to these variables and other variables, such as photosynthesis, are strongly influenced by the type of inoculum used [66,67,68], which demonstrated that the type of inoculum used influenced these characteristics. The present study highlights the high potential of soil yeasts and nanoparticles applied in a sodium alginate matrix as promising bioinoculants for improving plant production, which has notable implications for sustainable agriculture and food security.

Finally, the physiological and biochemical mechanisms by which NPs influence plant growth are diverse and complex; they include mechanisms such as interaction of biomolecules, modulation of hormonal signalling, stress induction, and plant defence response and also involve crucial processes such as absorption and translocation, where plants absorb NPs through the epidermis of the roots, tips, and lateral root junctions and are transported through the xylem to other parts of the plant; NPs enter mainly the leaves of the plant through cuticles, stomata, lenticels, hydathodes, stigmas, and trichomes and reach the phloem through translocation [56,69,70,71,72]. It is important to mention that this process is dependent on the type of NPs, size, soil conditions, and plant type, directly influencing photosynthetic processes. This process is dependent on the type of NPs, size, soil conditions, and plant type and all these factors directly influence photosynthetic processes [72].

## 4. Materials and Methods

### 4.1. Soil Collection and Characterisation

Reddish-brown soil was collected in the locality of Tranapuente, Carahue, La Araucanía region, southern Chile (38°41′27″ W 73°21′14.2″ W). Soil extraction was performed at the first 30-cm depth. The soil was sieved at 2 mm and mixed with sand at a 2:1 soil/sand *v*/*v* ratio. The mixture was autoclaved (121 °C for 20 min on 3 consecutive days). The soil properties included pH 5.4 (in water, 2:5 *w*/*v*), 12% organic matter, and nutrient concentrations of 15, 19, and 375 mg kg^−1^ of N, P, and K, respectively.

### 4.2. Determination of PGP Traits of Yeast Strains

Phosphate solubilisation activity was determined using two methods: (i) qualitatively using yeast isolates inoculated on Pikovskaya agar media and incubated at 30 °C for 7 days according to Amprayn et al. [33], and (ii) quantitatively according to Zaidi et al. [73], where yeasts were incubated in liquid media for 14 days. Siderophore production was determined by the change in the colour of solid blue Chromo-azurol S (CAS) medium according to Neilands [74]. The determination of 1-aminocyclopropane-1-carboxylate (ACC) deaminase activity was performed according to Nutaratat et al. [75]. To quantify the production of indole acetic acid, yeast isolates were inoculated in yeast extract peptone dextrose (YPD) media supplemented with 100 μg mL^−1^ tryptophan and incubated at 30 °C for 7 days. The colour intensity was determined at 530 nm on a Synergy HTR microplate spectrophotometer (BioTek, Winooski, VT, USA) using IAA as an external standard [75]. All analyses were performed in triplicate, and fresh YPD yeast cultures with an adjusted absorbance of 0.6 optical density were used to normalise the results in each case.

### 4.3. Physicochemical Characterisation of the α-Fe_2_O_3_-NPs

Capsules containing the soil yeasts and the α-Fe_2_O_3_ NPs used to perform these evaluations were manufactured according to the methodology described by Berríos et al. [76]. Commercial nanodust/iron oxide nanoparticles (α-Fe_2_O_3_, 99%) (SkySpring Nanomaterials, Inc., Houston, TX, USA) were characterised. Dynamic light scattering (DLS) was used to determine the hydrodynamic size, polydispersity index (PDI), and zeta potential using Zetasizer ZS90 119 equipment (Malvern Instruments Inc., Malvern, UK). The samples were previously diluted in deionised water to 0.01% to achieve a suitable scattering intensity. The measurements were performed in polystyrene/polystyrene cells (10 × 10 × 45 mm) at a fixed angle of 90°. The sample was placed directly into the measurement chamber of a particle electrophoresis instrument (DTS1079 cells; Zetasizer Nano series, Malvern, UK) to determine the zeta potential. The surface morphology and elemental composition were determined using a scanning electron microscope (SEM-STEM, SU3500) fitted with an electron-dispersive X-ray spectrometer (SEM/EDX) (Hitachi, Tokyo, Japan) at 15.0 kV, where the sample was placed on a carbon film-covered copper grid before analysis. The thermal properties of the α-Fe_2_O_3_-NPs were studied using a Simultaneous Thermal Analyser (STA) 6000 (Perkin Elmer, Waltham, MA, USA).

### 4.4. In Vivo Bioassays and Plant Growth Conditions

Commercial seeds of romaine lettuce cv. Bionda Degli Ortalani were used to perform the greenhouse experiments. Seeds were surface sterilised with 5% *w*/*v* sodium hypochlorite for 1 min, exposed to 70% ethanol, and washed with sterile water three times. The seeds were sown in polystyrene trays with a sterilised soil/sand mixture (2:1, *v*/*v*) and transferred after three weeks of germination to 0.5 L pots. Lettuce plants were grown in a greenhouse under controlled conditions, with a photoperiod of 16:8 h light/dark, 18/26 °C night/day, and 50/60% relative humidity. Plants were watered every two days, and fertilisation was applied via irrigation every 10 days using 50% Hewitt’s nutrient solution. Two yeast strains, *Candida guilliermondii* and *Rhodotorula mucilaginosa*, were used. Both strains correspond to the working group’s culture collection and were identified by Perez et al. [39]. A completely randomised factorial design composed of seven treatments and six replicates (N = 42) was used as an additional factor to the encapsulated Fe_2_O_3_-NPs in the presence and absence of Fe_2_O_3_-NPs. The following treatments were used: (T1), non-inoculated control; (T2), *Candida guilliermondii* with nanoparticles; (T3), *Candida guilliermondii* without nanoparticles; (T4), *Rhodotorula mucilaginosa* with nanoparticles; (T5), *Rhodotorula mucilaginosa* without nanoparticles; (T6), yeast consortium with nanoparticles; and (T7), yeast consortium without nanoparticles. The plants were harvested after 52 days.

### 4.5. Biomass Production

After 52 days, the plants were harvested. Leaves and roots of each plant were weighed fresh, placed in liquid nitrogen, and stored at −80 °C for 24 h. The leaf and root tissues were placed in 50 mL tubes and lyophilised. Samples were weighed again and stored in a dry and dark place until further analysis.

### 4.6. Photosynthesis Yield and Water Status

Measurements of photosynthetic and fluorescence characteristics were performed one day before harvest on the second youngest leaf of the three plants. The Targas-1 system (PP Systems, Amesbury, USA) was used to determine the following variables: (i) stomatal conductance (gs: nmol (H_2_O) m^−2^ s^−1^); (ii) internal CO_2_ concentration in leaves (Ci: µmol mol^−1^); (iii) photosynthetic rate (A: µmol (CO_2_) m^−2^ s^−1^); and (iv) water use efficiency (WUE: mmol CO_2_ mol^−1^ H_2_O). Fluorescence was measured using the FluorPen system (Photon System Instrument, Drasov, Czech Republic), where the quantum yield of photosystem II (QY: µmol CO_2_ µmol^−1^ absorbed photons) was evaluated.

### 4.7. Photosynthetic Pigments

Chlorophyll A and B were extracted from 100 mg of lyophilised plant tissue in 1.5 mL of extraction solvent (MeOH:water:formic acid 50:48.5:1.5 *v*:*v*:*v*), and the samples were filtered through 13 mm diameter Millex filters with 0.22 µm pore size nylon membrane (Millipore, Bedford, MA, USA). The absorbance of the supernatant was measured using an Epoch UV–Visible microplate spectrophotometer (BioTek, Winooski, VT, USA) at wavelengths of 663 nm and 645 nm for chlorophyll A and B, respectively. The pigment concentrations were calculated according to the formulas of Liententhaler et al. [77].

### 4.8. Statistical Analysis

All statistical analyses were performed in R version 4.2.1. After verifying the normality and homoscedasticity of the data, the datasets were subjected to two-way analysis of variance (ANOVA) with yeast inoculation and the presence of nanoparticles as sources of variation. For variables showing significant differences, means were compared using Tukey’s HSD multiple range test. A significance level of *p* < 0.05 was established for all cases using the R library “agricolae” v.1.3.5. The means ± standard errors are presented in bar charts, and significant differences between treatments are denoted by different lowercase letters. The dataset was also subjected to principal component analysis (PCA). Confidence ellipses (group means) of different treatments, inoculations, and the presence or absence of Fe_2_O_3_ nanoparticles were generated using the “FactoMineR” v.2.7 and “factoextra” v.1.0.7 packages.

## 5. Conclusions

It has been demonstrated in this study that the yeast strains *Candida guilliermondii* and *Rhodotorula mucilaginosa* have plant growth promoting effects. Specifically, *C. guilliermondii* showed promising results as a future inoculant, mainly due to its higher capacity to solubilise phosphate, supported by previous research suggesting an active role of yeasts in facilitating phosphate supply to plants. Therefore, there is a direct relationship between this process and increased plant growth, mainly due to the action of siderophores linked to increased iron availability to plants. The photosynthetic activity was crucial for the productivity and biomass of the lettuce crop; it was attributed to the process of stomatal opening and CO_2_ uptake creating a better photosynthetic efficiency, mainly in the application of *C. guilliermondii* with NPs. The encapsulation process of sodium alginate beads was remarkable, mainly for being an excellent carrier; a positive synergy between yeasts and Fe_2_O_3_-NPs was observed, thus improving plant growth; it also provided protection and improved the shelf life of the formulation, offering this encapsulation method a viable alternative for the application of PGP microorganisms in field conditions. These findings suggest the high potential of soil yeasts and nanoparticles encapsulated in sodium alginate matrices as effective bioinoculants to improve plant production. This approach has important implications for sustainable agriculture and food security, offering a promising alternative for improving crop growth and productivity, although in-depth research is required to evaluate their effectiveness in different types of crops and under limiting environmental conditions such as water stress.

## Figures and Tables

**Table 1:** In vitro determinations of the plant growth promoting activities of the two soil yeasts used in this study.

Yeast Species	Phosphate Solubilization (mg mL^−1^)		IAA (µg mL^−1^)	Siderophores	ACC Deaminase
	24 h	Day 14			
*Candida guilliermondii*	0.17 ± 0.00 c	1.53 ± 0.04 a	6.46 ± 0.02 b	+	+
*Rhodotorula mucilaginosa*	0.17 ± 0.01 c	0.69 ± 0.02 b	8.26 ± 0.02 a	+	+

Abbreviations: IAA, indole acetic acid; ACC deaminase, 1-aminocyclopropane-1-carboxyl-deaminase. Values were obtained as the mean of three replicates ± S.E. Means not followed by the same letter are significantly different according to Tukey multiple range test (*p* < 0.05).

## Data Availability

The data presented in this study are available upon request from the corresponding author.

## Supplementary Materials

The following supporting information can be downloaded at: https://www.mdpi.com/article/10.3390/plants13152042/s1, Table S1: Values of significance of variable analysed by two-way ANOVA, with a source of variation of yeast inoculation, presence of nanoparticles and the interaction of two variables.
