# Supplementary material for: Impact of Sodium Alginate-Encapsulated Iron Nanoparticles and Soil Yeasts on the Photosynthesis Performance of Lactuca sativa L. Plants
Source: Plants (Basel). 2024 Jul 25;13(15):2042. doi: 10.3390/plants13152042 (PMC11314604; doi:10.3390/plants13152042)
Supplement: Supplementary file 1 [file plants-13-02042-s001.zip › plants-3071054-supplementary.pdf]

Supplementary material

Table S1. Values of significance of variable analised by Two way ANOVA, with a source of variation of yeast inoculation, presence of nanoparticles and the interaction of two variables.
*p Value means *p < 0.05, ** p < 0.001, *** p < 0.0001. The variables described are the following:*

Variable	Yeast	Nanoparticle	Yeast : Nanoparticle
A	0.0029 *	0.0901	0.2283
Carotenoids	0.0228 *	2e-04 **	0.0132 *
Chlorophyll_A	0 ***	1e-04 **	1e-04 **
Chlorophyll_B	2e-04 **	3e-04 **	1e-04 **
Ci	0 ***	4e-04 **	0 ***
gs	0 ***	0.0012 *	0 ***
LFW	0 ***	0.2628	0 ***
QY	0.0012 *	0.2134	0.7587
RFW	0 ***	0.0131 *	0 ***
Total_Chlorophyll	1e-04 **	1e-04 **	1e-04 **
WUE	0.428	0.6378	0.0482 *

Fresh weight leaves (LFW); Fresh weight roots (RFW); Stomatal conductance (gs); Internal CO_2 concentration in leaves (Ci); Photosynthetic rate (A); Water efficiency (WUE); Quantum yield of photosystem II (QY); Chlorophyll A; Chlorophyll B; Total chlorophyll; Carotenoids.
